# The DAOA/G30 locus and affective disorders: haplotype based association study in a polydiagnostic approach

**DOI:** 10.1186/1471-244X-10-59

**Published:** 2010-07-29

**Authors:** Micha Gawlik, Ingeborg Wehner, Meinhard Mende, Sven Jung, Bruno Pfuhlmann, Michael Knapp, Gerald Stöber

**Affiliations:** 1 Department of Psychiatry and Psychotherapy, University of Würzburg, Füchsleinstraße 15, 97080 Würzburg, Germany; 2 Institute of Medical Biometry, Informatics and Epidemiology, University of Bonn, Sigmund-Freud-Str. 25, 53105 Bonn, Germany; 3 Department of Forensic Medicine, University of Würzburg, Lindleinstraße 15, 97080 Würzburg, Germany; 4 Coordination Centre for Clinical Trials, University of Leipzig, Härtelstraße 16-18, 04107 Leipzig, Germany

## Abstract

**Background:**

The DAOA/G30 (D-amino acid oxidase activator) gene complex at chromosomal region 13q32-33 is one of the most intriguing susceptibility loci for the major psychiatric disorders, although there is no consensus about the specific risk alleles or haplotypes across studies.

**Methods:**

In a case-control sample of German descent (affective psychosis: n = 248; controls: n = 188) we examined seven single nucleotide polymorphisms (SNPs) around *DAOA/G30 *(rs3916966, rs1935058, rs2391191, rs1935062, rs947267, rs3918342, and rs9558575) for genetic association in a polydiagnostic approach (ICD 10; Leonhard's classification).

**Results:**

No single marker showed evidence of overall association with affective disorder neither in ICD10 nor Leonhard's classification. Haplotype analysis revealed no association with recurrent unipolar depression or bipolar disorder according to ICD10, within Leonhard's classification manic-depression was associated with a 3-locus haplotype (rs2391191, rs1935062, and rs3916966; P = 0.022) and monopolar depression with a 5-locus combination at the *DAOA/G30 *core region (P = 0.036).

**Conclusion:**

Our data revealed potential evidence for partially overlapping risk haplotypes at the DAOA/G30 locus in Leonhard's affective psychoses, but do not support a common genetic contribution of the DAOA/G30 gene complex to the pathogenesis of affective disorders.

## Background

**Figure 1:**
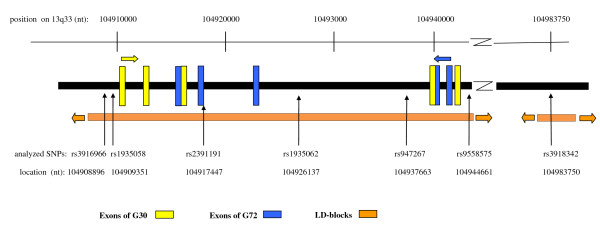
**Gene structure of G72/G30 on chromosome 13q33 and location of genotyped SNPs**. The G30 and G72/DAOA locus and locatiom of the analysed SNPs. G30 exons are marked with yellow, G72 with blue, LD-blocks with orange bars. Arrows indicate orientation on the chromosomal strand Chromosomal position (nt).

Based on whole-genome linkage data large proportions of the distal chromosome 13q (spanning < 50 cM) have been proposed as regions containing genes for schizophrenia, bipolar disorder, autism, anorexia and panic disorder [1,2]. In a systematic analysis targeting on a < 5 Mb segment at the distal region of chromosome 13q32-33, Chumakov et al. described two candidate genes for schizophrenia, *DAOA *and *G30*, overlapping on complementary chromosomal strands with opposite orientations (Figure 1) [3]. *DAOA *consists of five exons, spanning a region of < 25 kb, encoding 742 bp of putative mRNA, whereas *G30 *spans < 47 kb and the longest potential open reading frame encodes a 71-amino acid protein (Figure 1), [3]. Along with the discovery of G72/DAOA, a neurochemical cascade was launched that introduced G72/DAOA as part of the central glutamate system, which plays an essential role in the formation of memory, synaptic plasticity and neuronal development. G72 was renamed D-amino acid oxidase activator (DAOA) since initial experiments proposed G72 as potent interacting partner of D-amino acid oxidase (DAO) increasing NMDA transmission via D-serine oxidation [3].

Subsequently, numerous genetic association studies made the DAOA/G30 gene complex one of the most intriguing susceptibility loci for the major psychiatric disorders. The meta-analyses of published association studies supported weak, but significant genetic effects at the DAOA/G30 locus to schizophrenia for markers rs3916964 and rs2391191 or in Asian schizophrenia populations for rs947267 and rs778293, and in European populations for rs1421292 [4-6].

Association between bipolar affective disorder and the DAOA/G30 locus has first been reported in North American family-based samples at the intronic marker rs1935058, with significant association of the entire haplotype set [7]. Further studies missed replication, but pointed to further associated markers and haplotypes in case-control samples of US-European ancestry and from Germany, Poland, Finland and the United Kingdom [8-13]. The initial meta-analysis found the intronic single nucleotide polymorphism (SNP) rs1935062 the most promising marker (p = 0.0019) [4]. In a more recent meta-analysis based on four case-control association studies none of five single markers (used in more than one study) showed evidence of overall association, but all SNPs showed significant evidence for heterogeneity between study designs and study samples [6-9,11]. Regarding genotype-phenotype relations rs3918342 seemed related to psychotic features (persecutory delusions) in bipolar cases, and the ancestral G allele of rs2391191 (Arg30) to poor visuospatial performance in bipolars with mania and psychotic symptoms [10,13]. In a comparison of "core syndromes" of bipolar disorder and schizophrenia, the *DAOA/G30 *locus showed overall association to lifetime episodes of disturbed emotions more than to psychosis itself [11].

To further elucidate the genetic relevance of *DAOA/G30 *for affective disorders, we performed a haplotype based, case-control association study in a sample of German descent in a polydiagnostic approach including the criteria of ICD-10 and Leonhard's classification of endogenous psychoses. Leonhard's subphenotypes of bipolar and monopolar depression represent distinct clinical and nosological entities avoiding uncertainties by a switch from unipolar to bipolar depression during course of disease [14,15].

## Methods

Cases were recruited from the Department of Psychiatry, Psychosomatics and Psychotherapy at of the University of Würzburg. The sample encompassed 248 cases (154 males, 62%) with affective disorders with a mean age of 48.1 years (+ 15.7 SD) at recruitment. Diagnosis of recurrent unipolar depression with "somatic syndrome" (ICD10 F33.11-F33.3), was made in 129 cases and of bipolar affective disorder in 119 cases [14]. Age at first hospitalization was 42.7 (+ 16.1 SD) years and 35.9 (+ 12.8 SD) years in each group, respectively. In addition, cases had to fulfil diagnostic criteria of monopolar depression (n = 57) and manic depression (n = 191) according to Leonhard's nosology [15]. Diagnosis in differentiated psychopathology was made by repeated personal examinations of experienced psychiatrists (BJ, GS). The 188 volunteer control subjects (105 males, 59%) were recruited from the blood donor centre at the University of Würzburg at a mean age of 30.2 years (+ 10.7 SD). The preponderance of males in both samples avoided gender distortion in comparison of cases and controls. All subjects were unrelated and of German Caucasian descent. The Ethics Committee of the University of Würzburg had approved the study, and written informed consent was obtained from all subjects.

### Genotyping

Matching the DAOA/G30 locus with equally distributed markers we selected seven SNPs from published studies or the public databases http://http:www.ncbi.nlm.nih.gov/; http://genome.ucsc.edu: the intronic SNPs rs3916966 (M13), rs1935058, rs1935062, rs947267 (M18), the exonic marker rs2391191 (M15; coding for Arg39Lys), at the 5'-UTR of the DAOA/G30 gene complex rs3918342, and rs9558575 (Figure 1, Table 1) [3,7,10].

**Table 1:** Genotype distribution at the DAOA/G30 locus at chromosome 13q33 according to ICD 10

	Controls (n = 188)					Bipolar (n = 119)					Unipolar (n = 129)				
	Genotype					Genotype					Genotype				

SNP	11	12	22	MAF	HWE	11	12	22	MAF	P-value	11	12	22	MAF	P-value

rs1935058 (C/T)	32	77	79	0.38	0,09	23	54	42	0.42	0.29	26	56	47	0.42	0.30

rs947267 (C/A)	26	94	68	0.39	0,54	16	56	47	0.37	0.64	20	60	49	0.39	0.99

rs1935062 (A/C)	67	94	27	0.39	0,65	40	65	14	0.39	0.94	50	51	28	0.42	0.60

rs2391191 (A/G)	16	83	89	0.31	0,73	12	52	55	0.32	0.72	20	54	55	0.36	0.13

rs3916966 (A/C)	62	88	38	0.44	0,55	46	55	18	0.38	0.20	48	56	25	0.41	0.54

rs9558575 (G/T)	22	98	68	0.38	0,16	17	51	51	0.36	0.60	18	62	49	0.38	0.95

rs3918342 (C/T)	37	108	43	0.48	0,06	31	65	23	0.47	0.20	30	75	24	0.48	0.29

P-values according to Armitage's Trend Test

MAF: Minor Allele Frequency

HWE: Hardy-Weinberg Equilibrium

PCR for allelic discrimination was performed in a final reaction volume of 20 μl containing 20 ng genomic DNA and 10 μl of 2 × TaqMan^®^Universal PCR Master Mix (Applied Biosystems) and 1 μl of 20 × TaqMan™ SNP genotyping assay including fluorescent tags specific for the wild type allele and the variant allele. Marker amplification was performed in microtiter plates on Biometra thermocyclers (Whatman). PCR amplification conditions were according to the manufacturer's recommendation [10 min at 95°C followed by 15 sec at 92°C and 60 sec at 60°C for 40 cycles]. Allelic discrimination with endpoint detection of fluorescence was performed at 60°C on an ABI prism 7000 sequence detection system followed by analysis with an appropriate software package (Applied Biosystems). All genotype experiments were made at least in duplicate, with quality control of automated allele calling by two independent operators blind to phenotype (0% replicate error rate). Genotyping was completed for each marker in the total sample (no missing data). Positive and negative controls are included routinely in our genotyping experiments.

The exact test proposed by Weir was applied for Hardy-Weinberg equilibrium. To calculate the pairwise standardized linkage disequilibrium (LD) coefficient D' we used the program FAMHAP and the GOLD-software package [16,17]. Armitage's trend test was used to compare genotype distributions between cases and controls. The test hapcc implemented in the program FAMHAP was used to test all possible SNP combinations (consisting of up to seven SNPS) for their association with the disease [16,18]. FAMHAP also enables the calculation of a global/P/-value being corrected for multiple testing. The statistics on allele and genotype distribution were uncorrected. Power approximations were calculated with the program GenOdyPower [19].

## Results

Genetic evaluation of the core region of the DAOA/G30 gene complex was based on seven SNPs in a sample of 436 subjects (248 cases; Figure 1). Pairwise linkage disequilibrium (LD)-analysis between the markers confirmed that a single LD block encompasses all putative exons of the *DAOA/G30 *complex, from rs3916966 to the 35 kb upstream located rs9558575 at nt position 104944661 (Figure 1). SNP-marker rs3918342 is part of a distal LD block, in low LD with both rs947267 (D' = 0.28) and rs9558575 (D' = 0.33).

No single marker showed evidence of overall association with affective disorder (Table 1 and 2). Allele and genotype frequencies were not significantly different between cases and controls. The markers were in Hardy-Weinberg equilibrium (data not shown). We observed neither gender differences (data not shown) nor differences in the clinical subgroups according to ICD10 or Leonhard's classification (Table 1 and 2).

**Table 2:** Genotype distribution: Manic and monopolar depression according to Leonhard's classification

	Controls (n = 188)					Manic Depression (n = 191)					Monopolar Depression (n = 57)				
	Genotype					Genotype					Genotype				

SNP	11	12	22	MAF	HWE	11	12	22	MAF	P-value	11	12	22	MAF	P-value

rs1935058 (C/T)	32	77	79	0.38	0,09	36	86	69	0.41	0.30	13	24	20	0.44	0.25

rs947267 (C/A)	26	94	68	0.39	0,54	25	89	77	0.36	0.48	11	27	19	0.43	0.42

rs1935062 (A/C)	67	94	27	0.39	0,65	70	93	28	0.39	0.92	20	23	14	0.45	0.31

rs2391191 (A/G)	16	83	89	0.31	0,73	24	78	89	0.33	0.48	8	28	21	0.39	0.10

rs3916966 (A/C)	62	88	38	0.44	0,55	75	85	31	0.38	0.16	19	26	12	0.44	0.96

rs9558575 (G/T)	22	98	68	0.38	0,16	27	82	82	0.36	0.53	8	31	18	0.41	0.48

rs3918342 (C/T)	37	108	43	0.48	0,06	48	109	34	0.46	0.12	13	31	13	0.50	0.75

P-values according to Armitage's Trend Test

MAF: Minor Allele Frequency

HWE: Hardy-Weinberg Equilibrium

Regarding unipolar depression or bipolar disorder according to ICD10, permutation tests for best marker combinations and best single markers did not reach statistical significance (Table 3). Gender specific combinations did not appear. Within Leonhard's classification manic-depression was significantly associated with a 3-locus haplotype (rs2391191, rs1935062, and rs3916966; P = 0.022), whereas monopolar depression was associated with a 5-locus combination, containing SNPs of the *DAOA/G30 *core region, at P = 0.036 (Table 3).

**Table 3:** Marker combinations at DAOA/G30 locus for association with disease in a polydiagnostic approach

Diagnosis according to ICD 10	best marker combination	Global P-value
Unipolar Depression	rs1935058, rs947267, rs1935062, rs2391191, rs3916966, rs9558575	0.06

Bipolar Disorder	rs1935058, rs1935062, rs2391191, rs3916966,	0.18

Diagnosis according to Leonhard	best marker combination	

Monopolar Depression	rs1935058, rs947267, rs2391191, rs3916966, rs9558575	0.036

Manic Depression	rs1935062, rs2391191, rs3916966	0.022

Global P-values according to the analysis with FAMHAP, adjusted for multiple testing.

Best marker combination with the smallest unadjusted P-value.

## Discussion

Although positive linkage findings for psychiatric disorders at chromosome 13q and previous genetic association studies consider the DAOA/G30 gene complex a robust candidate for schizophrenia and affective disorder, there is no consensus about the specific risk alleles or haplotypes across studies [1,20]. In our case control study on 436 subjects, individual alleles in the gene complex were not significantly associated with affective disorder, neither subdivided according to ICD10 nor to Leonhard's classification [14,15]. Our negative findings on individual markers, thus, corroborate the data of a recent case-control study in a Scottish population on narrowly defined bipolar affective disorder, a family-based association study of US-European trios with DSM III-R and DSM IV bipolar I and schizoaffective bipolar type, and of a recent comprehensive meta-analysis on bipolar samples [6,12,21]. In addition, multilocus analyses failed to identify associated haplotypes in unipolar and bipolar depression (Table 3). In Leonhard's subtypes of affective psychoses, however, manic-depression showed a potential association with a 3-locus haplotype spanning ~90 kb, whereas monopolar depression was associated with a 5-locus haplotype in the core gene complex.

Our analysis of the LD structure of the *DAOA/G30 *complex confirms and extends data of earlier studies that the proximal LD block encompasses all putative exons of *DAOA/G30*, reaching from SNP rs3916966 to the 35 kb upstream located SNP rs9558575, which was for the first time included in an association analysis [7,8,22,11,24][HapMap project]. No haplotype-tagged SNP seems to appear. Associated SNPs on the distal block (i.e. rs3918342 or rs1421292) may, thus, be linked to regulatory or transcriptional elements of the *DAOA/G30 *complex.

To increase the complexity of the *G70/G30 *locus in affective disorder, an independent German sample had reported on a protective two marker haplotype rs3918342 and rs1421292 for bipolar disorder at the distal region which is located < 40-50 kb downstream to the predicted coding region of *DAOA/G30 *[9]. SNP rs3918342 was found to be particularly related to psychotic features (persecutory delusions) in this sample and in a Polish bipolar replication sample [10]. The risk haplotype rs3918342 and rs142129 appeared also to be associated with DSM IV recurrent major depression, whereas more proximal markers showed no association with disease [23]. Individual case-control and family based studies on bipolar disorder had reported various positive single marker and haplotype associations in European populations, only partially overlapping with the findings of a recent study on Asian populations which favoured rs778293 and a two-marker haplotype rs778294-rs778293 in the distal region for increasing risk for bipolar disorder [7,9,13,25].

In view of these divergent genetic findings it remains difficult to conclude whether these differences point to the genetic autonomy of individual phenotypes, represent a common genetic background for affective disorders. It confirms the importance of rigorous diagnostic categorization in affective disorders, the problem of sample recruitment strategies and the dilemma of suboptimal power. The strength of our strategy is the combination of an operational diagnostic approach with ICD-10 and Leonhard's categorical diagnostic approach maximizing homogeneous subgroups, though reducing power of the sample size. Table 4 exemplifies the different groups.

**Table 4:** Overview of different subgroups according to Leonhard and ICD 10

	ICD 10 bipolar (n = 119)	ICD 10 unipolar (n = 129)
Manic Depression according to Leonhard (n = 191)	119	72

Monopolar Depression according to Leonhard (n = 57)	0	57

Leonhard's conception displays some important differences compared to current conceptions of affective disorders. ICD and DSM have interpreted the diagnostic criteria of unipolar and bipolar disorders rather broadly. The concept of "endogenous depression" survived in the accessory term "somatic syndrome" (ICD10), and diagnosis of bipolar disorder is made by the genuine course knowing that 10-25% of unipolar patients switch to bipolarity in longitudinal studies [26-28]. Leonhard's subphenotypes of monopolar depression are characterized by distinct affective syndromes recurring in each episode with identical symptoms, whereas essential criteria for manic-depression are remitting course and bipolarity with a melancholic or manic basic syndrome, presence of mixed states or unipolar partial states with instability of mood. The melancholic core syndrome is characterized by depression, psychomotor and thought inhibition, varying depressive ideas, and somatic symptoms and the maniac core syndrome by elevation of mood, flight of ideas, pressure of speech, elevated self-consciousness, ideas of grandeur and goal-oriented activity. Psychotic features like persecutory delusions, incoherence of speech, mood-incongruent hallucinations generally do not fit with the diagnosis of manic-depression in the sense of Leonhard, but are indicative for cycloid psychoses or unsystematic schizophrenias[15]. Based on these diagnostic criteria, in manic-depression appears an excessive familial morbidity risk of 35.2% among first degree relatives compared to population controls (5.7%) and cycloid psychosis (10.8%) [29].

Our study inherits some limitations as it was directed at analyzing homogeneous subgroups though reducing power of the sample size. In comparison with the findings of Schumacher et al. our study has a power (at alpha = 0.05) of 22.3% for the monopolar depression, and of 44.4% for the manic depression according to Leonhard's classification [9]. According to ICD-10 the power is 35.2% for the bipolar depression and 36.8% for the unipolar depression. In addition we cannot exclude minor impacts by potential flipping of allele calling, although our LD-data are congruent to previous findings indicating if any a relative small effect.

Initially, DAOA was thought to be part of the central dysregulation of the glutamatergic N-methyl-D-aspartate (NMDA) receptor function, which is thought to be related to cognitive malfunction in patients with schizophrenia, depression and other neuropsychiatric disorders by effecting the long-term potentiation (LTP) pathway [3,30,31]. This was questioned by a recent study reporting better cognitive performance for risk allele carriers [32]. Although existing cDNA libraries proposed expression of DAOA in the amygdala, caudate nucleus, spinal cord, and testis, and DAOA and G30 mRNA expression seemed likely in post-mortem dorsolateral prefrontal cortex of patients with schizophrenia, no convincing reports regarding expression of native DAOA protein appeared [7,33]. In-vitro immunohistochemical analyses revealed some evidence that DAO and DAOA/G30 are both expressed in astrocytes of the human cortex, but binding experiments suggested DAOA more acting as a negative effector of DAO[34]. The DAOA protein product of 24-kDa was initially reported to localise at the Golgi apparatus but a more recent study demonstrated mitochondrial localisation of overexpressed DAOA [7,35]. Moreover, DAOA mRNA could not be detected in peripheral tissue samples and 13 brain regions of the human CNS using reverse transcriptase (RT)-PCR techniques and northern blotting, and the protein-protein interaction of DAOA and DAO failed reproducibility in recombinantly expressed protein experiments [36,35]. These findings did not support a putative function of DAOA as general regulator of DAO in the brain and in glutamatergic signalling either. The failure to detect expression within various tissues pointed to an extremely localised or tightly, developmentally regulated expression with a unique spatio-temporal role in human brain development, independent of an interaction with DAO [36]. This suggested that if the DAOA protein exists at all, it is expressed at such low levels that any physiological role is called into question [36]. For the second gene at the DAOA/G30 gene locus, no protein product could be verified thus far, suggesting that G30 is a regulator gene of unknown function or just a pseudogene. These physiological data further challenge a significant role of the DAOA/G30 gene complex for the aetiology of affective disorders. In addition to this genome-wide association studies provided no further evidence for an association of DAOA with schizophrenia or mood disorders challenging the previous positive findings [37].

## Conclusion

Despite the uncertainties regarding expression and function of the DAOA/G30 gene complex, the genetic association of the DAOA/G30 locus to neuropsychiatric disorders is considered robust, although identification of true causative variants is still lacking and associated alleles and haplotypes are not consistent across studies. Our findings point to partially overlapping risk haplotypes at the DAOA/G30 locus associated with Leonhard's affective psychoses, but do not support a common genetic contribution of the DAOA/G30 gene complex to the pathogenesis of affective disorders.

## Competing interests

The authors declare that they have no competing interests.

## Authors' contributions

MG carried out the molecular genetic studies and drafting of the manuscript, IW performed laboratory assays, SJ participated in the coordination of the study. BP and BJ participated in the diagnostic evaluation of the patients, MM and MK contributed the data-analysis, interpretation of the data and drafting of the manuscript, GS initiated and coordinated the study. All authors read and approved the final manuscript.

## Pre-publication history

The pre-publication history for this paper can be accessed here:

http://www.biomedcentral.com/1471-244X/10/59/prepub
